# Comparative Epidemiological and Clinical Outcomes on COVID-19 and Seasonal Influenza Hospitalized Patients during 2023

**DOI:** 10.3390/idr16050060

**Published:** 2024-08-23

**Authors:** Constantin-Marinel Vlase, Mariana Stuparu Cretu, Mihaela-Camelia Vasile, George-Cosmin Popovici, Manuela Arbune

**Affiliations:** 1 Doctoral Studies in Biomedical Sciences, “Dunarea de Jos” University Galati, 800008 Galati, Romania; constantin.vlase@ugal.ro (C.-M.V.); mihaela.vasile@ugal.ro (M.-C.V.); popovicigeorge1989@gmail.com (G.-C.P.); 2 Military Hospital “Dr. Aristide Serfioti” Galați, 800008 Galati, Romania; 3 Medical Department, “Dunarea de Jos” University Galati, 800008 Galati, Romania; 4 Clinic Emergency Children Hospital, 800487 Galati, Romania; 5 Infectious Diseases Clinic II, Infectious Diseases Clinic Hospital Galati, 800179 Galati, Romania; 6 Pneumology Department II, Pneumophtisiology Hospital Galati, 800170 Galati, Romania; 7 Clinical Medical Department, “Dunarea de Jos” University Galati, 800008 Galati, Romania; manuela.arbune@ugal.ro; 8 Infectious Diseases Clinic I, Infectious Diseases Clinic Hospital Galati, 800179 Galati, Romania

**Keywords:** viral diseases, SARS-CoV-2, influenza, vaccine

## Abstract

COVID-19 and influenza are highly contagious respiratory viral diseases and priority global public health concerns. We conducted a retrospective observational study of COVID-19 and/or influenza hospitalized cases, during 2023. We identified 170 influenza cases, 150 COVID-19 cases and 3 co-infections. Overall, 29.10% of patients had at least one COVID-19 vaccine dose and 4.6% received the seasonal Flu vaccine. The demographic data found older patients in the COVID-19 group and a higher index of the comorbidities, mainly due to chronic heart diseases, hypertension, and diabetes. Fever, chills, and rhinorrhea were more frequently related to influenza, while cough was prevalent in COVID-19. Antibiotics were more used in influenza than COVID-19, either pre-hospital or in-hospital. The mortality rate within the first 30 days from the onset of the respiratory infection was higher in influenza compared to COVID-19. We concluded that the COVID-19 clinical picture in hospitalized patients is changing to influenza-like symptoms. The evolution is variable, related to chronic comorbidities, but influenza had more frequent severe forms. All through 2023, due to poor vaccination rates, COVID-19 and influenza have continued to cause numerous hospitalizations, and a new strategy for efficient vaccinations is required.

## 1. Introduction

COVID-19 and influenza are viral respiratory illnesses with high contagiousness, constituting priorities for global public health. The public health restricted measures during the early pandemic in 2020–2021 resulted in an impact on the absence of the annual seasonal epidemic of most seasonal respiratory viruses [1]. The current epidemiology of the two infections reports annual recurrences in the case of influenza and a decreasing trend in the COVID-19 pandemic. The dynamics and epidemiological trends in COVID-19 and Flu in six WHO regions from January 2020 to March 2023 were retrospectively described by Wang Q. et al. by using a neural network model. They found that influenza activity was below 10% in the first COVID-19 pandemic year, but gradually increased during the declined Delta SARS-CoV-2 variant, although Delta continues to dominate, while Flu dominance alternated with COVID-19 during the Omicron variant. The activities of influenza and COVID-19 have shown a seesaw effect, due to their reciprocal suppression and competition [2]. Both influenza and coronaviruses are distributed to multiple hosts and co-circulation or co-infections allow genetic reassortment and recombination, resulting in new variants that are able to increase the cross-species transmission. Co-infection could be clinically more severe and the serum cytokines and chemokines could be related to clinical manifestation [3].

Hospitalized cases of COVID-19 continue to be reported, maintaining the risk of the emergence of new viral variants, which can cause waves of illnesses and deaths. Both viral etiological agents, SARS-CoV-2 and the influenza virus, are characterized by antigenic variations that occur over time. As the many viruses with high mutation rates, mutations could emerge at each position in the genome, evolving all possible nucleotides, and continuously produced genetic diversity allows viral escape from the host immunity [4]. Therefore, although COVID-19 and influenza are vaccine-preventable diseases, vaccine protection depends on the match between vaccine components and the antigenic configuration of the circulating viral strains. The symptomatology of influenza and COVID-19 is similar, with fever, dyspnea, dysphagia, rhinorrhea, and myalgia, and specific tests are necessary for differential diagnosis [5]. COVID-19-influenza co-infections have symptoms common to mono-infections, but the more severe progression carries a higher risk than mono-infections [6].

Despite numerous clinical trials, the antiviral treatment of COVID-19 has not yet been standardized, while influenza benefits from firm therapeutic recommendations [7,8]. This study aims to compare the clinical-epidemiological and evolutionary characteristics of hospitalized cases of influenza and COVID-19 in 2023.

## 2. Materials and Methods

We conducted a retrospective observational study of cases of influenza and COVID-19 admitted between 1 January 2023 and 31 December 2023 in a department of the Clinical Hospital for Infectious Diseases in Galati, located in southeastern Romania. We selected cases diagnosed according to the international classification of diseases from the hospital’s information system with codes U07.1 and J10.0, J10.1, and J10.8. During this period, all patients hospitalized with flu-like symptoms (fever, cough, sore throat, coryza, headache, malaise, myalgia, arthralgia, and/or hypoxemia, dyspnea, tachypnoea) were tested upon admission with rapid tests for both COVID-19 and influenza A/B. The diagnosis of influenza and COVID-19 was based on the case definitions for communicable diseases from the European Union [9].

Hospitalized cases were those complicated and associated with comorbidities at risk of severe progression, but not critical cases, as the hospital does not have an intensive care unit. Cases that worsened to critical forms were transferred to the tertiary emergency hospital. All hospitalized patients signed informed consent to use personal data for research purposes. We collected demographic data, medical history, vaccination history, symptomatology, clinical evolution, and complications during hospitalization. Thirty-day mortality post-hospitalization was assessed through telephone interviews with patients or contact persons listed in the informed consent signed upon admission, according to local procedures. We calculated the Charlson comorbidity index for each patient and compared the overall score value and frequency of the scored comorbidities [10].

The management of patients with COVID-19 and influenza followed the standard of care according to national protocols [11].

The statistical analysis used XL-Stat 2023.5.1. We employed descriptive statistical tests, calculating the frequency, the distribution shape of the values, and the central tendency parameters: mean, median, and standard deviation. We compared the relationship between the characteristics of the COVID-19 group and the influenza group, depending on the type of variables. We used the Mann–Whitney test to compare median differences for numerical variables, such as age, the duration from onset to hospitalization, and the length of hospital stay. Other variables used were ordinal data, compared using odds ratios and Fisher’s Exact Test. The Chi-Square Test of Independence was used to analyze if the association between two categorial variables is significant or not. A *p*-value of <0.01 was considered statistically significant. Cases with dual infection of COVID-19 and influenza were described distinctly but were not statistically analyzed.

This study received institutional approval from the Medical Council of Clinical Hospital for Infectious Diseases in Galati No. 4/2/23.05.2024 and No. 4/3/23.05.2024.

## 3. Results

**Figure A1:**
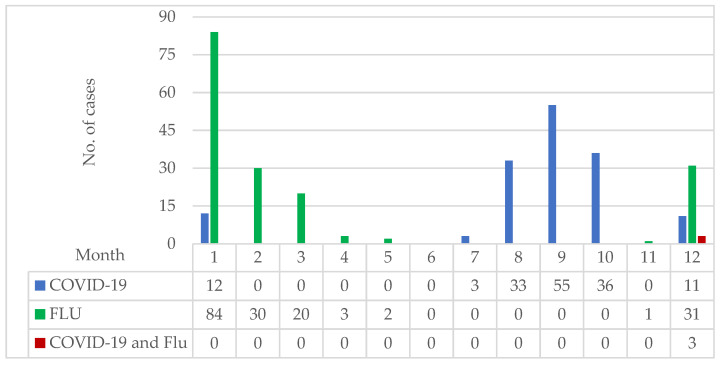
Monthly distribution of COVID-19 and Flu cases during 2023.

We identified 170 cases of influenza, 150 cases of COVID-19, and 3 cases of combined infections. By type of influenza virus, 90.5% had influenza A virus, and 9.4% had influenza B. Most hospitalized influenza cases were recorded in winter, while COVID-19 cases were predominantly concentrated in summer–autumn (Figure A1).

### 3.1. Vaccination Status

Out of the total group of patients, 29.10% had received at least one dose of the COVID-19 vaccine, only 4.6% had been vaccinated for seasonal influenza, and 3.4% had been vaccinated for both. Vaccine immunization did not protect 6.9% of the patients hospitalized with influenza and 30.66% of those hospitalized for COVID-19. Additionally, 12.66% of the COVID-19 group reported having had at least one previous episode of COVID-19 in their medical history.

### 3.2. Demographic Characteristics

The average age of patients with COVID-19 was 63.84 ± 18.7 years [min 9 years; max 90 years], significantly higher compared to 46.81 ± 25.59 years [min 1 year; max 94 years] for patients with influenza (Mann–Whitney test *p* < 0.001).

Other demographic characteristics highlighted a predominance of women and urban residency in both respiratory infections while there are differences in education and occupation. The COVID-19 group of patients were more educated but less professionally active (Table 1).

### 3.3. Co-Morbidities

The Charlson Comorbidity Index was significantly higher in patients with COVID-19 than those with influenza: 3.55 ± 2.42 vs. 2.17 ± 2.43 (Mann–Whitney test *p* < 0.001).

The most reported comorbidities were hypertension, chronic heart failure, cerebrovascular accident, and diabetes mellitus. The patients with COVID-19 were more frequently obese, hypertensive, diabetics, and had a history of cerebrovascular disease than patients with influenza (Table 2).

### 3.4. Clinical Characteristics

The median duration of symptoms from onset to hospitalization was 2 days, with variations between 1 and 10 days, for COVID-19 and influenza.

Most patients experienced fever and/or chills. The patients with influenza were more likely to present fever (OR = 2.43), chills (OR = 2.04), and rhinorrhea (OR = 3.37), while cough was significantly more frequent in patients with COVID-19 (OR = 2.62). Almost two-thirds of the patients reported headaches, both with COVID-19 and influenza. Dysphagia, myalgia, and asthenia had frequencies between 24% and 37%, but there were no significant differences between the patient groups. Other symptoms identified in both groups included arthralgia, dyspnea, vomiting, diarrhea, abdominal pain, vertigo, and syncope (Table 3).

Anosmia/Ageusia were reported in four patients with COVID-19 and in one patient with influenza. Other symptoms specified only for COVID-19 included dysphonia (3), confusion (2), balance disorders (2), epileptic seizure (1), and epistaxis (1).

### 3.5. Radiological Characteristics

**Figure A2:**
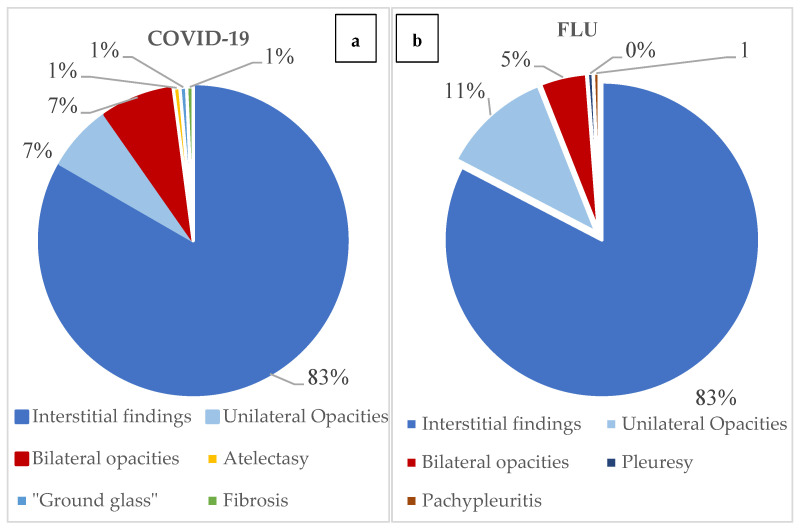
Comparative radiologic findings in COVID-19 (**a**) and FLU (**b**).

The main pulmonary radiological changes in COVID-19 were increased interstitial markings 83%, unilateral opacities 7%, and bilateral opacities 7%. Isolated cases reported ground-glass opacities, atelectasis, and pulmonary fibrosis. The distribution of radiological changes in influenza showed 83% interstitial changes, 11% unilateral opacities, 5% bilateral opacities, and isolated cases of pleurisy and pleuritis (Figure A2).

### 3.6. Antiviral and Antibiotic Treatment

Antivirals were administered to 96% of patients with influenza (Oseltamivir) and 79.3% with COVID-19 (Remdesivir).

Antibiotics were used prior to hospitalization in 39 influenza and 13 COVID-19 patients, and the difference was significant (OR = 3.11; Chi-Square test *p* < 0.001). During hospitalization, influenza patients also received systemic antibiotics, particularly third generation cephalosporins, more frequently than those with COVID-19 (18.2% versus 2% *p* < 0.001). During hospitalization, bacterial strains were isolated from only 4% of patients (related to six influenza and seven COVID-19): *Klebsiella pneumoniae* (3), *Escherichia coli* (3), *Moraxella catharalis* (2), *Staphylococcus aureus* (3), *Streptococcus pneumoniae* (2), Group B Streptococcus (1), *Acinetobacter baumannii* (1). Double bacterial isolates were found in two cases. *Staphylococcus aureus* and *Streptococcus pneumoniae* were identified in the sputum of a patient with influenza. Additionally, multidrug resistance screening from rectal swabs identified *Acinetobacter baumannii* and *Enterococcus faecium* in a patient with COVID-19. *Clostridioides difficile* diarrhea was present in nine patients.

### 3.7. Evolution, Complications

The median duration of hospitalization for both infections was 5 days, with variations between 1 and 15 days. Moderate forms of the disease predominated. Severe forms represented 10.7% in COVID-19 and 12.9% in influenza. There were no in-hospital deaths, but the condition worsened and 3 patients with COVID-19 and 11 patients with influenza were transferred to intensive care. The mortality rate within the first 30 days from the onset of the respiratory infection was higher in influenza compared to COVID-19 (Table 4). Most hospitalized cases were pneumonia. Complications such as sepsis, acute respiratory failure, and hepatitis were similar in both COVID-19 and influenza, but nephritis and coagulation disorders were more associated with COVID-19 (Table 4).

### 3.8. Characteristics of COVID-19 and Influenza Co-Infection

Three cases of concurrent infections of COVID-19 and influenza were reported in December 2023. These cases involved patients over the age of 68: two women and one man from rural areas, with heart failure and atrial fibrillation, unvaccinated for COVID-19 and influenza, and without confirmed prior COVID-19 infections. Fever was absent in all patients, while cough was the constant symptom. The three patients had respiratory failure-complicated pneumonic forms of the disease with complex radiological changes, including bilateral opacities (one case) and bilateral hyper-transparencies (two cases). All patients received both Remdesivir (3 days) and Oseltamivir (5–7 days) without any side effects and showed favorable progress (Table A1).

## 4. Discussion

The outbreak of the SARS-CoV-2 coronavirus pandemic in 2019 was characterized by explosive morbidity and mortality due to its unusually high transmissibility and virulence. Access to molecular diagnostic techniques enabled the identification of an extraordinary number of genomic sequences, leading to the emergence of distinct variants and phenotypes with specific characteristics regarding transmissibility, severity, and post-infectious immunity. The rate of substitutions in the virus’s molecular evolution, the rate of mutation through host-mediated genome replication errors, or the recombination of various viral genetic structures, which led to the emergence of hybrid variants, can all explain the diversification of SARS-CoV-2 during the pandemic [12].

The initial variant of SARS-CoV-2 was named Alpha, but within a few months, variants of concern (VOC) emerged and spread rapidly: Delta (2020–2021), Omicron BA.1, BA.2, BA.3, and BA.5 (2021), Omicron BA.5 (2022) [13,14,15].

In 2023, the circulation of Alpha, Delta, and Omicron strains was rare, with COVID-19 illnesses being attributed to the emergence of an Omicron subvariant EG5 (Eris). This variant, classified by the World Health Organization as a “variant of interest,” possesses a mutation responsible for evading post-vaccination or post-infection immunity and increasing transmissibility. It contributed to the rapid rise in cases in Romania in the second half of 2023, consistent with the trend observed in our study [16].

Upper respiratory tract symptoms characterized the prevalent variant in 2023, but elderly individuals (over 65 years) and those with immunosuppression may develop severe forms of pneumonia [17].

The circulation of the influenza virus maintains its seasonal character. In 2023, there were two epidemic seasons. From the 2022 to 2023 season, cases were concentrated in January, gradually decreasing until March, with isolated cases in April and May. The 2023–2024 season started late, with a single case in November and slightly increasing in December. In line with the European situation, Influenza Virus A was predominant [18,19]. 

The low vaccination rates can explain hospitalizations for the two respiratory infections amidst a general reluctance towards vaccination. A national study in Romania in 2023 showed that 28.4% of the general population was vaccinated against COVID-19, compared to 29.1% in our study, and 15.34% were vaccinated for seasonal influenza, compared to 4.6% of our hospitalized patients [20].

Comparative demographic data indicate a higher number of young patients hospitalized with influenza than COVID-19, consistent with active professions. Most hospitalized COVID-19 patients were over 65 years old, but there were also five pediatric cases. Influenza hospitalizations were distributed across all age groups, with the majority under 50 and 15.29% pediatric cases under age 15.

Co-morbidities more frequently associated with COVID-19 included obesity, hypertension, chronic heart failure, and diabetes mellitus, which have been known since earlier in the pandemic as risk factors for severe disease progression [21,22].

The symptoms of COVID-19 and influenza were overlapping, with no pathognomonic clinical features to differentiate between these viruses. A descriptive analytical large study from February 2020 to 2022 reported significant differences in symptoms among different variants of COVID-19, finding positive association with myalgia, cough, taste and smell disorders [23]. Compared to previous years, anosmia/ageusia were no longer indicators for suspected COVID-19 in our study, and they were rarely reported in 2023. Lehfeld A.S. et al. reported altered sense of smell and taste rates decreasing from 23% and 24% during the Delta phase to 7% and 8% in the Omicron phase [24].

The major radiologic findings in COVID-19 are airspace opacities, usually bilateral, peripheral, in the lower fields, but the radiological expression in our study was similar in COVID-19 and influenza, with 83% interstitial changes, followed by bilateral and unilateral opacities [25]. “Ground glass” images, suggestive of COVID-19, were identified in only one case, indicating a change in the clinical-radiological picture associated with new SARS-CoV-2 variants [26].

The use of antibiotics was more restricted for COVID-19 than influenza, similar with other reports [27]. However, *Clostridioides difficile* diarrhea was more frequent in COVID-19 patients. The greater vulnerability of COVID-19 patients to *Clostridioides difficile* may be due to the older age of the patients, but especially to the alteration of the intestinal microbiota, consequent to the inflammation of epithelial and stromal cells. Research has shown that SARS-CoV-2 enters cells by binding to the angiotensin-converting enzyme 2 receptor, abundantly found in the gastrointestinal tract [28].

The frequency of respiratory failure and sepsis were similar in both infections, but renal complications and coagulation disorders predominated in COVID-19 patients.

The mortality rate of COVID-19 hospitalizations has decreased worldwide compared to the pandemic’s beginning, but most studies indicate that the 30-day mortality rate remains higher than for influenza. In our study, the 30-day influenza mortality was slightly higher compared to other studies (4.71% vs. 3.7–3.8%) but also higher than COVID-19 mortality. The decrease in the death rate from COVID-19 in hospitalized patients may be due to post-vaccination or post-infection immunity, the decreased virulence of new SARS-CoV-2 variants, and the increased efficiency of patient care [29,30].

A large study compared epidemiology of respiratory viruses in pre-pandemic and during the COVID-19 pandemic. Pre-pandemic bi-annual peak seasonality of influenza was changed during the pandemic, to unclear seasonality, concomitant with decrease in the detection rate of influenza viruses. Moreover, the influenza positivity rates were higher in pediatric ages, either pre-pandemic or during the pandemic, while SARS-CoV-2 affected all ages over age 15 [31].

Co-infections between SARS-CoV-2 and influenza have been reported to be rare (<1%) [32]. The reciprocal circulation of both viruses allows the viral interference, which is characterized by a huge diversity, mainly in tropical regions. Viral competition is a transient inhibitory effect at the host level produced by a virus on a secondary infection by other viruses, mainly sustained interferon pathway activation [33].

In December, the concurrent circulation of influenza viruses and SARS-CoV-2 favored combined infections, which did not differ in clinical expression and severity compared to mono-infections. The incubation and contagiousness of COVID-19 are longer than those of influenza, making it difficult to differentiate between concurrent infections and sequential infections, with influenza superinfection in a patient with prolonged SARS-CoV-2 carriage. The therapy for co-infections is not standardized. Our clinical approach was the administration of Remdesivir for 3 days, combined with Oseltamivir for 5 to 10 days. Drug interactions between Remdesivir and Oseltamivir are not well known, but the estimated risk may be low due to the rapid metabolism of Remdesivir [34,35,36].

Post-respiratory virus outcomes were analyzed in the US, and compared the patients hospitalized with COVID-19 between 2020 and 2022 and seasonal influenza hospitalized between 2015 and 2019. In the COVID-19 group, higher mortality and increased risks of morbid consequences in all organ systems, excepting the lung, were reported. Longer term effects on the pulmonary system were experienced in influenza group [37].

Further research will bring more clinical data for better understanding the viral interactions and the post-infectious long-term consequences of influenza and COVID-19.

### Study Limitations

This study’s retrospective design did not allow for the standardized collection of certain data. The diagnosis was based on clinical data and the epidemiological context, with rapid test results being inadequate for confirming influenza according to the current methodology. Molecular investigations for identifying viral genotypes were unavailable for COVID-19 or influenza.

## 5. Conclusions

The number of COVID-19 cases in 2023 decreased, but the circulation of the virus continues, with the risk of emerging new variants that could cause waves of illnesses and deaths. COVID-19 and influenza continue to be causes of hospitalization due to insufficient vaccination prevention. Currently, the clinical and radiological pictures of the two viruses are overlapping, requiring specific tests for diagnosis. The evolution is variable, depending on age and associated chronic co-morbidities. Influenza more frequently had a worsening progression and a higher death rate than COVID-19. Intensifying health education efforts and extending vaccination are essential to limit the circulation of influenza and SARS-CoV-2, prevent severe forms of infection, and prevent the emergence of new viral variants with increased virulence.

## Figures and Tables

**Table 1:** Demographic characteristics of the patients hospitalized with COVID-19 and Flu.

		COVID-19 (N1 = 150)		Influenza (N2 = 170)		Odds Ratio	Chi-Square Test (*p*)
		n1	%	n2	%		
Gen	male	61	40.7%	74	43.5%	1.124	0.604
	female	89	59.3%	96	56.5%		
Living area	urban	90	60%	99	58.2%	0.929	0.748
	rural	60	40%	71	41.7%		
Education	illiterate	8	5.3%	31	18.2%	0.172 1.343 1.325	<0.001 0.407 0.295
	4 years	30	20%	30	11.8%		
	8 years	48	32%	48	25.3%		
	≥12 years	64	42.7%	76	44.70%		
Occupation	employed	25	16.7%	41	24.1%	0.406 3.362	0.002 <0.001
	retired	96	64%	64	37.6%		
	unemployed	29	19.3%	65	24.1%		

**Table 2:** Comparation of co-morbidities related to COVID-19 vs. Flu.

	COVID-19 (N = 150)		FLU (N = 170)		OR	CI 0.95	Fisher’s Exact Test (*p*)
	n1	%	n2	%			
Obesity *	58	38.7%	46	27.1%	1.69	1.06; 2.71	0.026
Hypertension	66	44%	51	30	1.83	1.15; 2.89	0.013
**Charlson Co-morbidities**							
MI	4	2.67%	2	1.18%	2.30	0.43; 12.17	0.570
CHF	26	17.3%	23	13.6%	1.33	0.72; 2.44	0.443
PVD	1	0.67%	4	2.35%	0.27	0.03; 2.19	0.224
CVA	20	13.3%	6	3.50%	4.17	1.73; 10.07	0.002
Dementia	7	4.67%	6	3.53%	1.33	0.44; 4.05	0.814
COPD	10	6.67%	8	4.71%	1.44	0.55; 3.74	0.604
CTD	2	1.33%	1	0.59%	2.28	0.21; 23.83	0.906
Peptic ulcer disease	5	3.33%	1	0.59%	5.82	0.86; 39.44	0.161
Liver disease	15	10%	11	6.47%	1.60	0.71; 3.59	0.343
Diabetes mellitus	38	25.3%	15	8.82%	3.50	1.88; 6.51	<0.001
Hemiplegia	5	3.33%	3	1.76%	1.91	0.46; 7.98	0.589
CKD	7	4.67%	8	4.71%	0.99	0.35; 2.80	1.198
Solid tumor	8	5.33%	4	2.35%	2.33	0.71; 7.67	0.269
Leukemia	1	0.67%	2	1.18%	0.56	0.05; 6.08	1.093
Lymphoma	2	1.33%	0	0	-	-	-
AIDS	6	4%	5	2.94%	1.37	0.41; 4.57	0.829

**Legend**: * Obesity defined by the body mass index (BMI) ≥ 30 kg/m^2^; MI: Myocardial infarction; CHF: Chronic heart failure; PVD: Peripheral vascular disease; CVA: Cerebrovascular accident; COPD: Chronic obstructive pulmonary disease; CTD: Connective tissue disease; CKD: Moderate-to-severe chronic kidney disease.

**Table 3:** Comparative clinical features of hospitalized COVID-19 vs. Flu during 2023.

	COVID-19 (N = 150)		FLU (N = 170)		OR	CI 0.95	Fisher’s Exact Test (*p*)
Fever	87	58%	131	77.1%	2.43	1.5; 3.9	<0.001
Chills	39	26%	71	41.8%	2.04	1.2; 3.2	0.003
Headache	50	66.7	59	65.3	1.06	0.6;1.6	0.795
Myalgia	45	30%	64	37.6%	1.40	0.8; 2.2	0.149
Arthralgia	14	9.33%	27	15.9%	1.83	0.9;3.6	0.110
Sore throat	37	24.7%	48	28.2%	1.20	0.7; 1.9	0.552
Rhinorrhea	10	6.67%	33	19.4%	3.37	1.65; 6.88	0.001
Cough	62	41.3%	36	21.2%	2.62	1.6; 4.2	<0.001
Dyspnea	24	16%	15	8.8%	1.96	0.9; 3.87	0.070
Vomiting	13	8.67%	28	16.5%	2.07	1.0; 4.1	0.053
Diarrhea	14	9.3%	7	4.1%	2.39	0.9; 5.9	0.097
Abdominal pain	5	3.3%	1	0.6%	0.17	0.0; 1.16	0.161
Chest pains	6	4%	6	3.5%	1.13	0.3; 3.6	1.050
Asthenia	41	27.3%	44	25.9%	0.92	0.5; 1.5	0.866
Vertigo	9	6%	10	5.8%	0.97	0.3; 2.4	1.14
Lipothymia	7	4.67%	3	1.76%	0.366	0.0; 1.3	0.243

**Table 4:** Outcome and complications of COVID-19 vs. Flu in hospitalized patients (2023).

	COVID-19 (N = 150)		FLU (N = 170)		OR	CI 0.95	Fisher’s Exact Test (*p*)
	n1	%	n2	%			
Outcome							
Mild	24	16%	29	17.1%	0.92	0.5; 1.67	0.919
Medium	112	74.7%	119	70%	0.79	0.4; 1.3	0.421
Severe	16	10.7%	22	12.9%	0.83	0.4; 1.6	0.651
Aggravation	3	2%	11	6.47%	3.38	0.9; 11.5	0.088
Deaths—30 days	4	2.67%	8	4.71%	1.80	0.5; 6.0	0.511
Complications							
Sepsis	1	0.7%	1	0.6%	1.13	0.0; 18.2	1.437
Pneumonia	141	94%	151	88.8%	0.50	0.2; 1.1	0.148
Respiratory failure	15	10%	20	11.8%	0.83	0.4; 1.6	0.747
Hepatitis	14	9.33	22	12.9%	1.44	0.7; 2.9	0.400
Nephritis	35	23.3%	13	7.65%	3.67	1.9; 7.0	<0.001
Hypercoagulation	39	26%	2	1.18%	29.51	10.8; 80.2	<0.001
*Cl. difficile* Diarrhea	8	5.3%	1	0.6%	9.52	1.7; 53.4	0.022

## Data Availability

The data presented in this study are available upon request from the corresponding author. The data are not publicly available due to privacy issues.

## Appendix A

**Table A1:** Summary of Co-infection COVID-19 and Flu (3 patients) in 2023.

	Patient 1	Patient 2	Patient 3
Gender	F	F	M
Age (years)	68	83	70
Vaccine C/F	0/0	0/0	0/0
Duration from onset to admission (days)	1	4	3
Fever	No	No	No
Clinical appearance	Cough Asthenia	Cough Asthenia Chills Myalgia	Cough Chills Myalgia
Co-morbidities	CHF Obesity Depression	CHF CKD Obesity hypertension	CHF COPD
X-rays findings	Bilateral opacities	Interstitial enhancement Pachypleuritis Bilateral hypertransparency Perihilar bilateral fibrosis	Unilateral opacity Bilateral hypertransparency Bronchiectasis
Complications	RF Nephritis	RF Hypercoagulation	RF
Antivirals (days)	Remdesivir (3) Oseltamivir (6)	Remdesivir (3) Oseltamivir (10)	Remdesivir (3) Oseltamivir (5)
Hosp. length (days)	11	9	5
Outcome (discharge)	Improved	Improved	Asymptomatic

Legend: F—female; M—male; Vaccine C/F—COVID-19 or Flu vaccine; CHF—chronic heart failure; CKD—chronic kidney disease; COPD—chronic obstructive pulmonary disease; RF—respiratory failure.
